# Micro-RNAs Predict Response to Systemic Treatments in Metastatic Renal Cell Carcinoma Patients: Results from a Systematic Review of the Literature

**DOI:** 10.3390/biomedicines10061287

**Published:** 2022-05-31

**Authors:** Martina Monti, Susanna Lunardini, Igino Andrea Magli, Riccardo Campi, Giulia Primiceri, Francesco Berardinelli, Daniele Amparore, Daniela Terracciano, Giuseppe Lucarelli, Luigi Schips, Matteo Ferro, Michele Marchioni

**Affiliations:** 1 Department of Medical Oral and Biotechnological Science, “G. d’Annunzio” University of Chieti and Pescara, 66100 Chieti, Italy; martinamonti@live.it (M.M.); susanna.lunardini@gmail.com (S.L.); iginomagli@gmail.com (I.A.M.); giulia.primiceri@gmail.com (G.P.); francescoberardinelli@hotmail.com (F.B.); luigi.schips@unich.it (L.S.); michele.marchioni@unich.it (M.M.); 2 Unit of Urological Robotic Surgery and Renal Transplantation, Careggi Hospital, University of Florence, 50134 Florence, Italy; riccardo.campi@gmail.com; 3 Department of Oncology, School of Medicine, San Luigi Hospital, University of Turin, Orbassano, 10124 Turin, Italy; danieleamparore@hotmail.it; 4 Department of Translational Medical Sciences, University ‘Federico II’, 80138 Naples, Italy; daniela.terracciano@unina.it; 5 Department of Emergency & Organ Transplantation—Urology, Andrology & Kidney Transplantation Unit, University of Bari, 70121 Bari, Italy; giuseppe.lucarelli@inwind.it; 6 Division of Urology, European Institute of Oncology, IRCCS, 10060 Milan, Italy

**Keywords:** advanced renal cell carcinoma (mRCC), metastatic, immunotherapy, tyrosine kinase inhibitor, miRNA, plasmatic, urinary, exosomal, liquid biopsy

## Abstract

Locally advanced or metastatic renal cell carcinomas (mRCCs) account for up to 15% of all kidney cancer diagnoses. Systemic therapies (with or without surgery) represent gold standard treatments, mostly based on tyrosine kinase inhibitors in association with immunotherapy. We provide an overview of the current knowledge of miRNAs as predictors of treatment resistance. A systematic review of the literature was carried out in January 2022 following the PICO methodology. Overall, we included seven studies—four testing plasmatic miRNAs, two exosomal miRNAs, and one urinary miRNA. A total of 789 patients were included (354 for plasmatic miRNAs, 366 for urinary miRNAs, and 69 for exosomal miRNAs). Several miRNAs were tested within the included studies, but six plasmatic (miR9-5-p¸ miR-192, miR193-3p, miR-501-3p¸ miR-221, miR-376b-3p) one urinary (miR-30a-5p), and three exosomal (miR-35-5p, miR-301a-3p, miR-1293) were associated with resistance to systemic treatments or treatment failure in mRCC patients. Results showed a fair accuracy of these biomarkers in predicting treatment resistance and overall survival. However, to date, the biomarkers tested have not been validated and their clinical uses are not recommended. Nevertheless, the literature results are encouraging; future large clinical trials are warranted to validate the effectiveness of these tools in clinical decision-making.

## 1. Introduction

Renal cell carcinoma (RCC) is one of the most common solid malignancies in both genders; it includes a heterogeneous group of tumors derived from renal tubular epithelial cells, accounting for 3% [1] of all cancers worldwide, with over 400,000 new cases each year [2].

Kidney cancer might have a heterogeneous clinical presentation, from small localized tumors to very aggressive metastatic diseases; prognosis is strongly related to various tumors and host characteristics [3]. RCC, when diagnosed early, is potentially curable by surgery; however, about one-fifth of patients have metastases at their diagnoses and another 20% of patients might have relapses and develop metastases during the follow-up after curative treatments [4]. Despite treatment advances, metastatic RCC (mRCC) patients have poor prognoses with a median survival about 20 months, approximately [5].

Standard treatment for mRCC is based on cytoreductive nephrectomy and a combination of systemic treatments, including targeted therapies and novel immunotherapeutic agents [6]. Current guidelines recommend cytoreductive nephrectomy (with or without metastasectomy) in patients who have good prognoses or in select patients at intermediate risk [6].

Risk classification is mainly based on prognostic factors identified by Motzer [7] and Heng [8]. However, those scores have shown several limitations, such as the absence of metastatic burden evaluation and poor accuracy [9]. For these reasons, new models that include the number of metastasis sites have been developed (with fair accuracy) [9]. Moreover, the clinical utilities of these models have been questioned [10]. Therefore, the research looked at new non-invasive biomarkers to better stratify patients.

Micro-RNAs (miRNAs), among the others, have been investigated due to their availability in bodily fluids, such as blood and urine. Few studies have shown an association between specific miRNA expressions and systemic treatment efficacy in patients with mRCC [11].

Therefore, their inclusion in available scoring systems might improve the classification accuracies and effectiveness. To date, there are a lack of comprehensive reviews that summarize the available knowledge on the role of miRNAs in mRCC treatment resistance development. This review offers an overview of the current evolution in the screening of circulating miRNAs as predictors of therapeutic responses in patients with mRCC undergoing systemic or local treatments and evaluates their potential for patient identification and stratification.

## 2. Materials and Methods

### 2.1. Searching Strategy

We performed a systematic literature review of scientific studies (published until May 2022) based on liquid biopsies in metastatic RCC. The review protocol was registered at the International Platform of Registered Systematic Review and Meta-Analysis Protocols and is available online (https://inplasy.com/inplasy-2022-4-0086/; last access 30 March 2022). The bibliographic search was performed using PubMed (Medline database, https://pubmed.ncbi.nlm.nih.gov/; last access 30 April 2022). The following terms were used: “(metastatic renal cell carcinoma) AND ((miRNA) OR (liquid biopsy))”. For the Medline search, the following filters were used: English language, human species, and full-text availability. Two independent authors (M.M. and S.L.), who ascertained the titles and abstracts, selected the studies that could be eligible for full-text evaluation. The full-text evaluation was performed by two independent authors (M.M. and L.S.). Conflicts were solved by a senior author (M.M.). Letters to the editor and reviews of the literature were excluded but the list of references was ascertained for articles of interest.

### 2.2. Selection of Studies

Studies were identified and selected according to the preferred reporting items for systematic reviews and meta-analysis (PRISMA) criteria [12] and the population, intervention, comparator, outcome (PICO) methodology [13]. The population consisted of mRCC patients receiving systemic treatments (intervention). Only studies that compared patients based on miRNA expression (plasmatic, exosomal, and urinary) were deemed eligible (comparator). The main outcomes of interest were the treatment response and overall survival (OS). For studies presenting scores or prognostic models, the accuracy reported as the area under the curve (AUC), or the C-index, were also considered.

### 2.3. Studies and Patient Characteristics

For each selected article, the following features were abstracted: lead author, country of origin, study period, and main outcomes of interest. Moreover, for each miRNA, we noted if it was down- or over-expressed, its functional activity, and the response to TKI therapy. Since only a few papers accomplished our inclusion criteria, we did not perform any quantitative analysis of the included results. Instead, we proposed a critical review of the current literature.

## 3. Results

### 3.1. Study Selection

**Figure 1:**
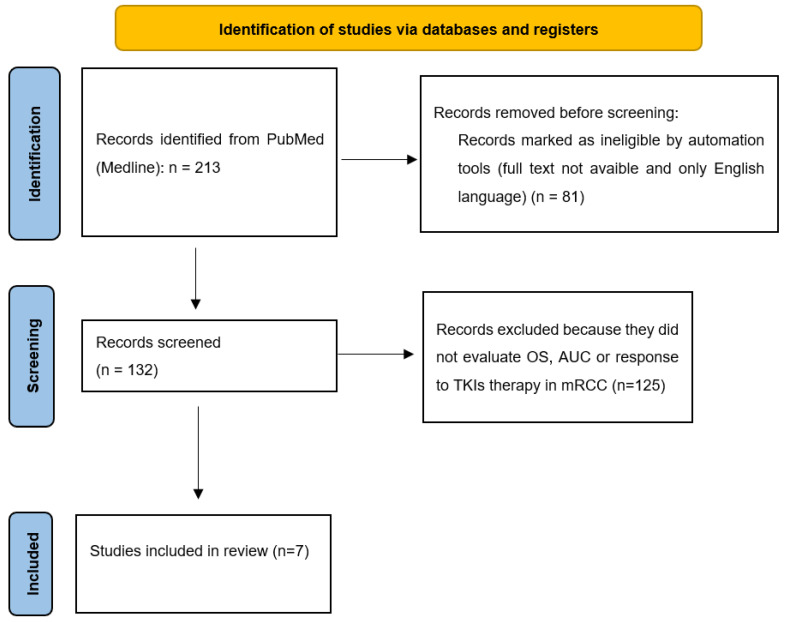
The PRISMA flowchart graphically depicts the selection of the study flow.

The PRISMA flow chart was used to summarize the process of study selection; it is shown in Figure 1. A total of 213 abstracts and titles were initially identified; after the initial screening, 81 studies were discarded because they were not compliant with our inclusion criteria. After full-text ascertainment, seven articles were selected in our systematic review. The PRISMA checklist is provided as supplementary material.

### 3.2. Liquid Biopsy and miRNAs

miRNAs are small non-coding single-stranded RNA molecules, about 21–25 nucleotides in length; their main functions involve regulating gene expression in a variety of manners [14]. miRNAs could act intracellularly or be actively secreted by cells and contribute to intercellular or cell–tissue communication. They bind the 3′-UTR regions on mRNAs and promote the translational repression or degradation based, respectively, on their imperfect or perfect complementarities with target mRNA [15].

It has been well documented that miRNA alterations can contribute to tumorigenesis; in fact, miRNAs can be actively loaded into extracellular vesicles by cancer cells in order to support cancer development and spread [16]. These molecules are expressed on the surfaces of multiple tumor markers and they can be exploited, i.e., isolation via the affinity purification technique and selecting them among all other extracellular vesicle populations in the fluid [16]. This is evidence that circulating miRNAs reflect the host’s response and there is an interaction between the tumor and its microenvironment.

miRNAs released from tumors or stromal cells are remarkably stable in human biofluids, including plasma and serum, due to their packaging in membranous vesicles (including exosomes, microvesicles, and apoptotic bodies) and their association with RNA-binding proteins [17].

In addition to the most commonly studied plasmatic miRNAs, a potential source of a tumor biomarker derived from urinary miRNAs [18] could provide alternative non-invasive procedures to the blood serum or plasma, as well as exosomes, small nanoparticles secreted into the plasma or urine from various types of cells [18].

### 3.3. Plasmatic miRNAs

Overall, four included studies tested the role of plasmatic miRNAs on therapy response prediction in mRCC patients. A total of 354 patients were included and 6 miRNAs were examined (Table 1).

The study conducted by Teixeira et al. [19] investigated the circulating expression levels of miR-221 as potential biomarkers for RCC detection and their influence on overall survival in comparison to other clinical covariates, such as gender, age, histology, clinical stage, and grade. The C-index was used to compare the predictive ability of the association of well-known prognostic variables with the miR-221 circulating expression levels. A C-index > 0.5 is considered as a threshold for good prediction ability [19]. Overall, 77 subjects were recruited for the study, 43 with diagnoses of RCC as cases (74.4% males). Another 34 healthy subjects, without known histories of cancer (58.8% males), were randomly recruited as controls. Only 14% of patients had metastases at diagnosis. Among the RCC patients, those with metastasis at diagnosis presented higher circulating expression levels of miR-221 than patients with no metastasis (*p* = 0.001) [19]. In addition, a significantly lower overall survival was observed in patients with higher expressions of miR-221 (48 vs. 116 months, respectively; *p* = 0.024). Models predicting overall survival, which included both clinical features and miR-221 expression levels, had meaningfully higher accuracy than those just including clinical features (C-index: 0.910 vs. 0.800) [19].

Ralla et al. [20] investigated the role of miR-9-5p in resistance against TKIs in comparison to other clinical covariates, namely age, sex, tumor stage, Fuhrman grade, metastatic, and resection status. The authors included 60 patients with diagnoses of mRCC who underwent radical nephrectomy from 2000 through 2011 and were subsequently treated with TKIs as first-line therapy; more specifically, 51 (85%) were treated with sunitinib, 6 (10%) with pazopanib, and 3 (5%) with sorafenib [20]. Overall, 32 patients (59.2%) were metastatic at diagnosis and were treated using cytoreductive nephrectomy, while the rest were classified as pN0/M0. All patients were treated with TKIs only after evidence of metastases. Overall, 41 (68.3%) patients were classified as responders [20]. The authors investigated several miRNAs; however, a statistically significant association with the TKI non-responder status was shown only with the overexpression of miR-9-5p and with the under-expression of miR-489-3p [20]. Nonetheless, when researchers tested the accuracy of each miRNA predicting the non-responder status, only miR-9-5p showed a fair accuracy. Indeed, the AUC was 0.83 (95%CI: 0.71–0.91) and it correctly classified 73% of patients. Not even the combination of multiple miRNAs had better results than miR-9-5p alone [20]. However, the authors showed a meaningful advantage in terms of accuracy when miR-9-5p was included in a model with clinical features. Such a comprehensive model had a higher AUC 0.89 (95%CI: 0.78–0.91) vs. just clinical features (AUC: 0.72, 95%CI 0.59–0.83) or the model including only miR-9-5p (AUC: 0.83; 0.71–0.91) [20].

Another study, conducted by Gamez-Pozo et al., investigated the predictively of models including miRNA expression for sunitinib resistance to therapy [21]. Overall, 38 metastatic RCC patients who received first-line treatment with sunitinib were included in a prospective observational multicenter study [21]. Blood samples were taken before and after two weeks from treatment initiation, and micro-array assays were performed, while promising miRNA candidates were retested with a real-time quantitative polymerase chain reaction (qPCR) [21]. Patients were stratified into two groups, namely poor and prolonged responders to TKI therapy, respectively, with median time to progression of 14 and 24 months, respectively. The authors showed that different models, including one or more miRNAs (up to four), had accuracies from about 60% to over 90% predicting poor responses and from over 80% to about 100% when predicting prolonged responses to sunitinib treatment [21]. In the poor response group, the median time to progression was 3.5 months and the overall survival was 8.5 months, whereas in the prolonged response group these values were 24 and 29.5 months, respectively. Best model candidates were also tested with RT-qPCR and results were virtually the same with microarrays, validating the authors’ findings. When such models were compared to MSKCC risk classification for predicting overall survival, the model including miR-192, miR-193-3p, and miR501-3p showed good ability at identifying poor responder patients and fair accuracy in predicting overall survival. Conversely, no association between MSKCC classes and OS was found [21].

According to a study conducted by Kovacova et al., miR-376b-3p can be used to predict sunitinib therapy response in metastatic tumors [22]. The authors included 179 mRCC patients. Therapeutic response was assessed with the response assessment criteria in solid tumors (RECIST) and the progression-free survival interval (PFS) was used to define the therapeutic response [22]. Patients were classified into three groups: (I) patients with primary resistance (PFS < 5 months), (II) patients with intermediate responses (PFS > 5 but <12 months), and (III) patients with long-term responses (PFS > 12 months). For the study, two cohorts were selected: a development cohort, which included 25 good response cases and 22 poor response cases, and a validation cohort, which included 132 cases [22]. Within the latter, a significantly higher proportion of intermediate response patients (*n* = 42) was included. First, high-efficiently miRNA microarray profiling was performed within the development cohort and a group of candidate miRNAs was selected [22]. Second, candidate miRNAs were validated within the validation cohort using real-time quantitative PCR. Out of 158 miRNAs (65 downregulated, 93 upregulated), miR-376b-3p was independently validated and was differentially expressed in tumors of patients with primary resistance versus long-term response (*p* < 0.0002) [22]. Gradually decreasing levels of miR-376b-3p were observed from those who were long-responders to those classified as non-responders. miR-376b was able to predict response to sunitinib therapy and identify long-term responsive patients vs. non-responders with a sensitivity of 83% and a specificity of 67% (*p* = 0.0002, AUC = 0.758) [22].

### 3.4. Exosomal miRNAs

Overall, two studies in the literature tested the role of exosomal miRNAs on the prediction of therapy response in mRCC patients and three miRNAs were examined (Table 2). He et al. [23] did not report the study data for privacy and ethical restrictions. A total of 69 patients were tested in the second study [24]. He et al. [23] suggested that miR-31-5p transported by extracellular vesicles participated in the development of resistance to the multi-targeted receptor TKI sorafenib.

The authors reported higher circulating levels of miR-31-5p in treatment-resistant patients after treatment compared to before. The authors also tested this hypothesis in in vitro and in vivo models. Interestingly, treatment-sensitive cell lines acquired resistance to treatment through the transmission of miR-31-5p by extracellular vesicles. Pathways for the development of such resistance were investigated and it was shown that miR-31-5p has a direct target to the homolog MutL 1 (MLH1) [23]. MLH1 protein is a component of a system of seven DNA mismatch repair proteins that work in sequential steps to start DNA mismatch repair in humans. Defects in MLH1 are associated with the microsatellite instability observed in different kinds of cancers, including TKI resistance in mRCC [23,25].

Dias et al. [24] enrolled 69 ccRCC patients who were divided into two groups: group A was composed of 32 patients with localized disease (who underwent surgical intervention); Group B was composed of 37 patients with metastatic disease. Blood from Group A patients was collected three times during the study: before surgery and 4 and 12 months after surgery; conversely, Group B patients underwent blood collection only once [24]. The authors observed differences in miRNA levels when samples obtained after surgery for localized tumors were compared to metastatic patient samples. Metastatic samples presented higher levels of hsa-miR-301a-3p (*p* = 0.026) and lower levels of miR-1293 (*p* = 0.004) [24]. Moreover, levels of has-miR-1293 and has-miR-301-3p were associated with overall survival in this subgroup of patients. Interestingly, lower levels of hsa-mir-25-3p were associated with shorter overall survival [24]. Moreover, patients with localized ccRCC presented variations in the patterns of plasmatic EV-derived miRNAs after tumor removal, with a decrease of hsa-miR-25-3p, hsa-miR-126-5p, hsa-miR-200c-3p, and hsa-miR-301a-3p, and an increase of hsa-miR-1293 [24].

### 3.5. Urinary miRNAs

Unfortunately, there are few studies on urinary miRNAs in mRCC; indeed, we found only one significant study [26]. A total of 366 patients were included and only 1 miRNA was examined [26]. This study found that higher miR-30a-5p independently predicted metastatic dissemination and survival (Table 3).

In urine sediment samples, miR-30a-5p levels identified cancer in both multicenter cohorts included in the study. In the first prospective cohort, significantly higher miR-30a-5p levels were found in the urines of 53 ccRCC patients compared to the 57 asymptomatic controls (AC), identifying malignancy with 83% sensitivity and 53% specificity, providing an overall accuracy of 67% (AUC of 0.684) [26]. In validation, a retrospective cohort of 171 ccRCC patients and 85 AC were included. The miR-30a-5p levels were significantly higher in ccRCC patients compared to AC [26]. Furthermore, miR-30a-5p levels identified ccRCC with 63% sensitivity, 67% specificity, an accuracy of 63%, and an AUC of 0.67. Moreover, higher miR-30a-5p levels independently predicted metastatic dissemination and survival. In the only available study on urinary miRNAs, the proportion of patients with metastatic disease was 10% [26].

## 4. Role of miRNAs on Cancer Pathophysiology

miRNAs are a family of small non-coding single-stranded RNAs that regulate a wide array of biological processes, including tumorigenesis [27]. The increased or decreased expressions of these molecules play key roles in cellular microenvironments, modulating important cell processes involved in carcinogenesis and cancer progression [28]. In fact, they have the ability to influence cell proliferation, cell motility, and metastatic processes, and can modulate the cell cycle progression and the acquisition of resistance to antineoplastic therapies [19].

Noteworthily, the activity of each miRNA varies based on different tissues. Moreover, each miRNA can target multiple mRNAs, while mRNA could be target for several miRNAs. Therefore, the same miRNAs may play different (even opposite) roles in regulating drug resistance in different cancer cells [29].

In our case, for example, some miRNAs were reported to be differentially expressed in various kinds of cancers. In fact, the deregulation of miR-221 was observed in several different malignancies, such as leukemia [30], as well as in colorectal [31], bladder, prostate, and kidney cancers [32]. These small molecules play key roles in the regulation of several pathways. The most important pathways involved are those mediated by p27 and the TRAIL pathway. These pathways are involved in cell cycles and apoptosis regulation [32]. More recent studies have hypothesized the roles of mir-221 and mir-222 in regulating pre-metastatic microenvironments in patients who develop liver metastases from colorectal cancer [32]. However, these pathways and interactions are complex and not fully understood [32].

Controversial results have been found for the miR-9-5p role. This molecule represents the main transcripts of MIR9-1, MIR9-2, and/ MIR9-3 genes, located on chromosomes 1, 5, and 15 [20]. Researchers showed a proapoptotic effect of MIR9-1 in patients with lung cancer and suppression of cell proliferation mediated by ubiquitin-like with PHD and ring finger domains 1 (UHRF1) [33]. Similarly, other studies showed that miRNA-9 overexpression enhances cisplatin sensitivity in hepatocellular cancer by mediating cell proliferation through EIFA5A2 epithelial–mesenchymal transition regulation [34]. Conversely, miR-9-5p expression seems to be associated with higher TKI resistance in mRCC patients [20]. These results suggest that the effect of this, as well as other miRNAs, could be tumor-dependent. As highlighted above, miRNAs might regulate several different processes and the cancer–host interactions could also be affected by their expressions.

Results were also controversial for miR-31-5p. Indeed, its overexpression has been associated with clinical responses in patients with cervical cancer; conversely, high levels of the same miRNAs were associated with more advanced (and worse) colorectal cancer prognoses [35]. Such duality is due to the dual actions of these miRNAs that have both onco-suppressor and oncogenic roles [35]. Indeed, miR-31 targets both oncogenic pathways, such as RAS, and suppresses the activity of other pathways, such as the androgen-dependent pathway [35]. The exact molecular mechanisms of action of these miRNAs have not been completely elucidated.

This is a further demonstration of the fact miRNAs can play key roles in tumorigenesis, tumor progression, and metastasis. This depends on the fact that they act as oncogenes and downregulate tumor suppressor genes; in fact, they are frequently overexpressed in cancer, while tumor suppressor miRNAs downregulate oncogenes and are often under-expressed in cancer [36].

## 5. Discussion

Our literature review summarizes current knowledge about miRNAs in mRCC treatment failure. Our review presents several interesting results that corroborate the possible roles of miRNAs as biomarkers in mRCC patients.

First, all included studies reiterate the role of miRNAs as key molecules in RCC development and progression. Our systematic review of the literature corroborates studies shthat models based on miRNA expression and traditional clinical–pathological features have higher accuracies than those based solely on clinical or tumor features [37,38,39]. Such consideration is of great importance when timing cytoreductive nephrectomy. Recently the SURTIME study showed the importance of delayed cytoreductive nephrectomy in patients diagnosed with mRCC, classified as intermediate risk [40]. However, a proportion of these patients might be refractory to systemic treatment and experience an early progression after TKI treatment. In this subgroup of patients, which might account for one-fifth of patients [41], upfront cytoreductive nephrectomy could be a better approach, avoiding treatment delay and the development of symptoms.

Second, several treatment combinations were approved in the mRCC setting, such as the immunotherapy agents alongside a TKI [42]. However, the response to TKI or immunotherapy agents could be different in specific patient categories, some patients could have larger advantages via the combination of two immunotherapy agents, as shown in intermediate or high-risk patients [40]. miRNAs could be useful to distinguish patients with a higher response to immunotherapy instead of TKIs. This hypothesis was corroborated by Kovacova et al. [22], who showed an association between miR-376b-3p expression and the response to sunitinib therapy. Similarly, Zhang et al. [43] showed that primary RCC cells with high levels of miR-183 were less sensitive to the cytotoxicity induced by NK cells. Therefore, miRNAs could be used to identify patients who should be treated by immunotherapy or TKIs based on their specific “miRNA signature”. Indeed, some tumors might be more resistant to the immune system activity and be refractory to immunotherapies; conversely, others might be resistant to targeted therapies [22,43]. According to a study conducted by Zhai et al. [44], miR-452-5p is downregulated after sunitinib treatment and is upregulated in metastatic renal cell carcinoma (RCC) tissues, both in in vivo and in vitro experiments. In this study, miR-452-5p could be a potential therapeutic target for kidney cancer [44]. miR-452-5p binds directly to SMAD4 (proteins that modulate the activity of transforming growth factor-beta ligands) and suppresses its expression, regulating the SMAD4/SMAD7 signaling [44]. The results suggest that miR-452-5p might be involved in sunitinib repressing RCC invasion and metastasis. In conclusion, miR-452-5p could be a potential therapeutic target for sunitinib and is associated with a poor prognosis of mRCC [44]. This might demonstrate that peripheral blood miRNA signatures could be employed to personalize therapy for advanced RCC, both in the selection of existing drugs and in the development of new drugs. The study conducted by He et al. [23] suggested that miR-31-5p may act as a tumor suppressor in RCC by inhibiting proliferation, migration, and invasion through cyclic-dependent kinase targeting 1.

Third, few studies investigated the role of exosomal protein markers to assess their diagnostic and prognostic potential as liquid biomarkers for clinical routines [45]. According to a study conducted by Jingushi et al. [45], Azulfidine (AZU1) is one exosomal protein to consider [45]. Tumor-derived exosomes are rich in this protein and the exosomes may play functional roles in driving metastatic dissemination. Despite initial reports, exosomal miRNAs or proteins have not yet been thoroughly studied as potential biomarkers for metastatic disease. Another study (by Fujii et al. [46]) investigated the relationship between miR-224 expression and patient prognosis in 108 ccRCC patients. Clinicians stratified RCC patients into two groups based on the median exosomal miR-224 expression levels. Those with high expressions of exosomal miR-224 had significantly shorter progression-free survival (PFS), cancer-specific survival (CSS), and overall survival (OS), compared with the low-level expression group [46]. The AUC was 0.83 for cancer progression, 0.86 for cancer-specific mortality, and 0.86 for overall survival [46]. High exosomal miR-224 expression was a significant independent risk predictor related to PFS, CSS, and OS in the multivariate analysis, adjusting for important covariates, namely, gender, age, stage, Fuhrman grade, and lymph vascular invasion [46]. However, this miRNA has not been tested in the mRCC.

Fourth, urinary miRNA might provide a non-invasive alternative to blood serum or plasma. Urine is easier to collect and could also allow for daily patient monitoring [47]. Urine could be considered promising in monitoring metastatic progression in RCC patients thanks to the possibility of quantifying miRNA’s increase or decrease, predicting the mRCC response to systemic treatment as a possible application in the near future in clinical practices. One interesting example of urinary miRNA that might show potential clinical utility is miR-210, overexpressed in ccRCC patients [47]. Urine samples from 75 patients with ccRCC and 45 control subjects (healthy patients) were analyzed; the levels were found to be importantly higher in patients with RCC with an AUC of 0.76, 57.8% sensitivity, and 80.0% specificity. In addition, the expression levels of urinary miRNA-210 significantly decreased in the patients a week after surgical intervention, as confirmation of the tumoral origins [47]. Unfortunately, the role has not been investigated in metastatic patients.

To the best of our knowledge, this is the first comprehensive review of the literature that specifically focuses on the role of miRNA as a possible predictor of survival in patients with mRCC. Our review highlights the potential of miRNA use in clinical practices. Future studies might focus on the clinical use of these biomarkers to stratify patients before treatment. As discussed above, the use of miRNAs might provide useful information about individual responses to different types of treatments, helping with patient selection for immunotherapy, targeted therapy, or a combination of systemic treatments. In addition, the role of these small molecules in an inflammatory response and immune system regulation will warrant a deeper knowledge of the relationship between the tumor and host. It is the authors’ opinion that efforts should be made to find new urinary miRNAs that could work as biomarkers for diagnoses, treatment choices, and monitoring. Indeed, urinary miRNAs are easy to collect; their variations could be evaluated on an (almost) daily basis, warranting strict follow-ups with patients and tailored treatments. Future studies in clinical and pre-clinical settings should investigate this possibility and aim to develop inexpensive and standardized assays that could be used in clinical and research practice.

However, our study was not devoid of limitations. First, most of the studies included had small sample sizes that reduced the generalizability of findings. Moreover, most of the included studies did not report any external validations of the findings. In addition, due to the lack of standardization and the limited number of studies included for each miRNA, we could not perform a quantitative synthesis of available results in the literature.

## 6. Conclusions

To date, no serum, urinary, or exosomal biomarkers tested have been validated and their use in clinical routines is not recommended. Nevertheless, literature results are encouraging, and future large clinical trials are warranted to test the effectiveness of these tools in clinical decision-making. Future studies, including basic research, should investigate the possibility of developing inexpensive and standardized assays for urinary miRNAs. Indeed, these biomarkers have the potential to be easy and daily available markers that are useful for treatment choices and monitoring.

## Figures and Tables

**Table 1:** The main characteristics of studies investigating the effects of plasmatic miRNAs in patients diagnosed with renal cell carcinoma.

First Author (Year)	Country (Study Design)	miRNA	Patients’ Characteristics	Main Results
Texeira et al. (2014)	Portugal (Retrospective)	miR-221	77 RCC patients	miR-221 levels were higher in the plasma of metastatic patients than patients with no metastasis; high expression correlated.
Ralla et al. (2018)	Germany (Retrospective)	miR9-5-p	60 mRCC patients treated with nephrectomy + TKIs (32 M+ at diagnosis)	A total of 41 (68.3%) patients were classified as responders. Overexpression of miR-9-5p and under-expression of miR-489-3p statistically significantly associated with the TKI non-responder status.
Gomez-Pozo et al. (2012)	Spain (Prospective)	miR-192 miR193-3p miR-501-3p	38 mRCC patients treated with sunitinib	miR-192, miR-193-3p, and miR501-3p have the ability to identify poor responder patients to TKI therapy.
Kovakova et al. (2019)	Czech Republic (Retrospective)	miR-376b-3p	179 mRCC	Gradually decreasing levels of miR-376b-3p were observed in those who were long-responders to those classified as non-responders. miR-376b predicted the response to sunitinib therapy and identified long-term responsive patients vs. non-responders.

TKI: Tyrosine kinase inhibitors.

**Table 2:** The main characteristics of studies investigating the effect of exosomal miRNAs in patients diagnosed with renal cell carcinoma.

First Author (Year)	Country (Study Design)	miRNA	Patients’ Characteristics	Main Results
He et al. (2020)	China (Retrospective)	miR-31-5p	-	Higher miR-35-5p. Increased resistance to TKI therapy (sorafenib).
Dias et al. (2020)	Portugal (Prospective)	miR-301a-3p miR-1293	69 RCC (37 M+)	Localized disease vs. metastatic; higher miR-301a-3p and lower miR-1293.

**Table 3:** Main characteristics of studies investigating the effects of urinary miRNAs in patients diagnosed with renal cell carcinoma.

First Author (Year)	Country (Study Design)	miRNA	Patients’ Characteristics	Main Results
Outeiro-Pinho et al. (2020)	Portugal (Prospective and retrospective multicenter)	miR-30a-5p	Prospective cohort: 53 ccRCC, 57 AC *. Retrospective cohort: 171 ccRCC, 85 AC *.	Higher miR-30a-5p levels independently predicted metastatic dissemination and survival

* Asymptomatic controls.

## Data Availability

Not applicable.

## Supplementary Materials

The following supporting information can be downloaded at: https://www.mdpi.com/article/10.3390/biomedicines10061287/s1. PRISMA 2020 Checklist [48].
